# Co-production of a feasibility trial of pacing interventions for Long COVID

**DOI:** 10.1186/s40900-023-00429-2

**Published:** 2023-03-30

**Authors:** Grace M. Turner, Christel McMullan, Olalekan Lee Aiyegbusi, Sarah E. Hughes, Anita Walker, Felicity Jeyes, Yvonne Adler, Amy Chong, Lewis Buckland, David Stanton, Elin Haf Davies, Shamil Haroon, Melanie Calvert

**Affiliations:** 1 Institute of Applied Health Research, University of Birmingham, Birmingham, UK; 2 Centre for Patient Reported Outcomes Research, University of Birmingham, Birmingham, UK; 3 NIHR Surgical Reconstruction and Microbiology Research Centre, University of Birmingham, Birmingham, UK; 4 NIHR Birmingham Biomedical Research Centre, University of Birmingham, Birmingham, UK; 5 NIHR Birmingham-Oxford Blood and Transplant Research Unit (BTRU) in Precision Transplant and Cellular Therapeutics, University of Birmingham, Birmingham, UK; 6 National Institute for Health and Care Research (NIHR) Applied Research Collaboration West Midlands, Birmingham, UK; 7 Birmingham Health Partners Centre for Regulatory Science and Innovation, University of Birmingham, Birmingham, UK; 8 Patient Partner, Centre for Patient Reported Outcomes Research, University of Birmingham, Birmingham, UK; 9 Aparito Ltd, Wrexham, Wales

**Keywords:** Long COVID, Post Covid-19 condition, Post-acute sequelae of SARS-CoV-2 infection (PASC), Coproduction, Pacing, Fatigue, Feasibility, Patient and public involvement

## Abstract

**Background:**

The high incidence of COVID-19 globally has led to a large prevalence of Long COVID but there is a lack of evidence-based treatments. There is a need to evaluate existing treatments for symptoms associated with Long COVID. However, there is first a need to evaluate the feasibility of undertaking randomised controlled trials of interventions for the condition. We aimed to co-produce a feasibility study of non-pharmacological interventions to support people with Long COVID.

**Methods:**

A consensus workshop on research prioritisation was conducted with patients and other stakeholders. This was followed by the co-production of the feasibility trial with a group of patient partners, which included the design of the study, the selection of interventions, and the production of dissemination strategies.

**Results:**

The consensus workshop was attended by 23 stakeholders, including six patients. The consensus from the workshop was to develop a clinical trial platform that focused on testing different pacing interventions and resources. For the co-production of the feasibility trial, patient partners selected three pacing resources to evaluate (video, mobile application, and book) and co-designed feasibility study processes, study materials and undertook usability testing of the digital trial platform.

**Conclusion:**

In conclusion, this paper reports the principles and process used to co-produce a feasibility study of pacing interventions for Long COVID. Co-production was effective and influenced important aspects of the study.

## Abstract (summary)

The World Health Organisation defines Long COVID as a condition which impacts people 3 months after they first had COVID-19. Some of the symptoms that characterise Long COVID symptoms include fatigue, breathlessness and brain fog. These symptoms have a major impact on people’s health and quality of life. Today, over 2 million people in the United Kingdom suffer from Long COVID and there is a lack of drugs and non-drugs treatment. However, some non-drugs treatments which aim to manage fatigue in other conditions, such as pacing, could be used with people with Long COVID. In this paper, we report how we co-produced a study which tested whether or not it is feasible for people who have Long COVID to use a pacing resource and report their symptoms using an electronic platform. After a meeting to review existing non-drugs treatments, the research team and a group of patient partners agreed on co-developing a clinical trial platform to test different pacing resources. The research team then met with the patient partners twice a week to co-design the study during which people with Long COVID will use the pacing resources and report their symptoms. They also co-designed the study documents and how to report its results. Co-producing a study with patient partners was effective and influenced important aspects of the study.

## Background

The high incidence of COVID-19 globally has led to a large prevalence of Long COVID, with the Office for National Statistics estimating there to be in excess of 2 million cases in the United Kingdom [1].The World Health Organisation (WHO) defined long COVID (or post COVID-19 condition, post-acute sequelae SARS-CoV-2 (PASC)) as “a condition which occurs in individuals with a history of probable or confirmed SARS-CoV-2 infection, usually 3 months from the onset of COVID-19 with symptoms that last for at least 2 months and cannot be explained by an alternative diagnosis” [2]. Long COVID is characterised by a wide range of symptoms 12 or more weeks following SARS CoV-2 infection, including fatigue, breathlessness and brain fog [2, 3]. These symptoms often have significant impacts on health, quality of life and work capability [4].

Despite the significant disease burden, there are a lack of pharmacological and non-pharmacological treatments for Long COVID. Existing non-pharmacological therapies, such as pacing, are based on expert consensus, with pacing being recommended by the WHO as an energy conservation technique to manage fatigue [5]. Pacing is defined as “energy management, with the aim of maximising cognitive and physical activity, while avoiding setbacks/relapses due to overexertion.” In other words, pacing is about knowing when to stop and rest by listening to and understanding one’s own body [6]. A systematic review of non-pharmacological treatments for post-viral syndromes, including Long COVID, found limited evidence and highlighted the urgent need for trials to assess the effectiveness of non-pharmacological therapies for Long COVID [7].

**Fig. 1:**
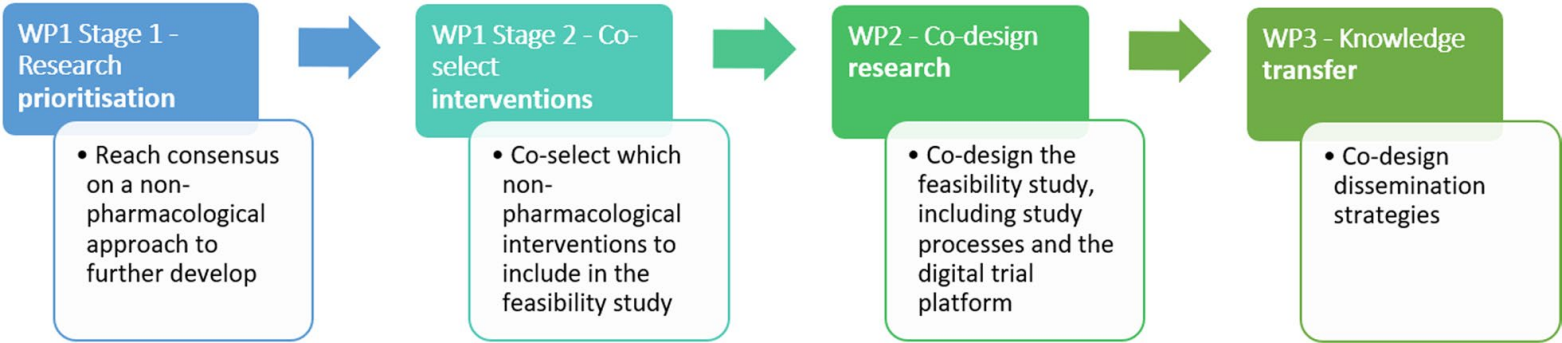
Summary of co-production

There is an urgent need to evaluate existing treatments for symptoms associated with Long COVID. However, there is first a need to test the feasibility of undertaking randomised controlled trials of non-pharmacological interventions for Long COVID, to inform the development of fully powered clinical trials and the trial platforms for delivering them at scale and pace. We aimed to co-produce a feasibility study of non-pharmacological interventions to support people with Long COVID (Fig. 1).

## Methods

The work reported is part of the Therapies for Long COVID (TLC) programme of research (National Institute for Health and Care Research (NIHR): COV-LT-0013) [8]. The key work packages and objectives are summarised in Fig. 1.

### Objectives

The objectives of coproduction with people with lived experience of Long COVID and other stakeholders for the TLC Study were to:

1. Support research prioritisation to reach consensus on a non-pharmacological approach to further develop to support people with Long COVID.
2. Co-select non-pharmacological interventions to include in the feasibility study.
3. Co-design the feasibility study, including study processes (such as recruitment) and the digital trial platform.
4. Co-design dissemination strategies to support knowledge transfer.

### Intervention remit

The scope of the research funding for this aspect of the TLC Study was to develop a feasibility study of non-pharmacological intervention(s) delivered through a decentralised digital clinical trials platform. The intervention needed to be non-pharmacological, and drugs or supplements were therefore not considered. The intervention needed to be delivered digitally through a platform called Atom5™, which was produced by a medical technology company Aparito Limited; a partner on the TLC Study. The Atom5™ app is a regulated (ISO13485, ISO/UEC 27001:2013 Accreditation, FDA CFR21 Part 11 compliant) software platform providing remote data collection and real-time patient monitoring through a person’s mobile device. It consists of two interfaces:

1. A clinician dashboard accessed via a web browser onto which research team members (Research Nurses) input patient and their trial information as part of users’ registration; manage users’ information; and undertake data downloads;
2. A patient-facing interface accessed via an app on Android/iOS devices onto which trial participants input their data.

The intervention could not be a Class II medical device as Atom5™ can provide feedback to research participants, but is not intended to provide medical advice or decisions.

1. Definition of co-production

In 2018 and 2019, the NIHR developed guidance aimed at improving the use of PPIE in research [9, 10]. In accordance with this guidance, we defined co-production as “an approach in which researchers, practitioners and the public work together, sharing power and responsibility”. As such, public contributors (people with lived experience of Long COVID) and stakeholders who were part of co-production were considered equal partners in the research, had joint ownership of key decisions and attended research meetings, where appropriate.

### Ethics

Ethical approval for the consensus workshop and feasibility trial was granted by the West Midlands -Solihull Research Ethics Committee (REC reference 21/WM/0203). Ethical approval was not required for other co-production activities with the patient and public involvement and engagement (PPIE) group.

### Work package 1

#### Aim: Intervention co-selection

##### Stage 1: research prioritisation

Potential non-pharmacological interventions to support people with Long COVID or similar conditions, such as Myalgic Encephalomyelitis/Chronic Fatigue Syndrome (ME/CFS) were identified through literature reviews [7, 11–13], relevant clinical guideline [14] and recommendations from people with lived experience of Long COVID from the TLC PPIE group, social media and Long COVID forums. In addition, as part of the TLC study, we conducted a systematic review to evaluate the effectiveness of non-pharmacological interventions for post viral syndromes, including Long COVID, as compared to either standard care, alternative non-pharmacological therapy, or placebo [7]. The outcomes of interest were changes in symptoms, exercise capacity, quality of life (including mental health and wellbeing), and work capability. Potential interventions were excluded if they were unable to be delivered digitally or there was a lack of evidence for their effectiveness on improving health and quality of life. Subsequently, four potential approaches to support people with Long COVID were proposed for prioritisation. These included interventions for breathwork and pacing, a decision support tool that maps Long COVID symptoms to recommended investigations and management, and a clinical trial platform to evaluate existing non-pharmacological interventions.

A consensus workshop was held to reach agreement on which of the above intervention approaches to further develop. Consensus workshop participants were purposively recruited to achieve a diverse mix of stakeholders, including people with lived experience of Long COVID, consultant physicians, general practitioners, public health experts, nurses, allied health professionals, academic experts, and regulators. Participants were recruited from known contacts and our PPIE group.

The workshop lasted one and half hours and was delivered remotely (via Zoom) to increase accessibility and reduce participant burden associated with travel, in-person attendance and to minimise COVID-19-related risk. The workshop structure was based on an adapted James Lind Alliance ranking and consensus process [15]. Participants were first sent pre-reading material, which included a summary of the workshop’s remit and potential intervention approaches. During the workshop, a short presentation was delivered on the background, remit and potential intervention approaches being considered. This was followed by small group discussions in three facilitated break-out groups lasting 30 min. Participants then did a first round of voting for their preferred intervention. There was then a whole group discussion after voting results had been shown to the group, lasting 20 min. The meeting closed with a second round of voting in which participants again voted for their preferred intervention.

##### Stage 2: co-selection of interventions

Based on feedback from people with lived experience of Long COVID who participated in the consensus workshop, potential pacing interventions were systematically identified from a survey distributed on social media, by contacting consensus workshop participants, and through searches on Twitter (using the #pacing hashtag), the Apple app store, Google Play, YouTube, and the Google search engine. A list of pacing resources for Long COVID and other long-term conditions was compiled. Patient partners and the research team produced a reduced list based on initial review, which was examined in detail by patient partners. A series of group meetings were held with the patient partners, facilitated by two researchers (GT and CM), to reach a final consensus on the interventions to include in the feasibility study.

### Work package 2: co-design feasibility trial

#### Aim: to co-design the feasibility trial with the PPIE group

A co-production group of five people with Long COVID co-designed the feasibility study with the research team. These members (who we will refer to as ‘patient partners’ in the rest of this paper) were recruited from Long COVID support groups. Patient partners were non-hospitalised and had been living with Long COVID for two years when they joined the TLC study. The co-production group attended virtual meetings twice a week, lasting one hour each, between March to July 2022. The first meeting of each week was attended by the patient partners and several members of the research team, which had a focused agenda covering operational aspects of the research project. This usually included a short break to enable patient partners with fatigue and brain fog, which are common symptoms in long COVID [4], to participate throughout the duration of the meetings. The second meeting of each week included patient partners and one researcher (CM), which allowed a more flexible discussion about the research project, patient experience, and peer support. Frequencies of the meetings were decided by the patient partners and there was no obligation to attend every meeting. Informal training was delivered whereby information and education was provided at relevant timepoints, such as explaining ethics processes. Roles within the coproduction group included co-design of research processes (such as recruitment strategies), participant facing study materials (such as participant information sheets) and testing the Aparito Atom5™ study platform, including downloading the app, testing the randomisation process, completing questionnaires on the app, and navigating through the app.

### Work package 3: knowledge transfer

#### Aim: to select knowledge transfer activities

The co-production group (from work package 2), provided feedback on planned knowledge transfer activities for academic outputs and co-designed public engagement and dissemination outputs for a general audience.

### Work package 4: evaluation and reflection

#### Aim: to evaluate PPIE involvement in co-production process

The team agreed on guiding principles for co-production at the start of the project. Throughout the process, the research team and patient partners informally reflected and provided feedback on co-production. Towards the end of the process, we used a more structured approach to evaluation whereby structured discussions were held with patient partners and the research team in separate meetings to reflect on their experiences. For people who could not attend these meetings, feedback was provided by email.

## Results

### Work package 1: intervention co-selection

#### Stage 1: research prioritisation

The consensus workshop was attended by 23 stakeholders, including six people with Long COVID (Table 1). In addition, ten people from the TLC research team and Aparito attended as facilitators or observers.

**Table 1:** Demographic characteristics of the consensus workshop participants (n = 23)

	N (%)
Sex	
Male	5 (21.7)
Female	18 (78.3)
Age	
18–25 years	1 (4.3)
26–35 years	7 (30.4)
36–45 years	8 (38.8)
46–55 years	4 (17.4)
56–65 years	3 (13.0)
Ethnicity	
White (White British, White Other)	15 (65.2)
Black African, Caribbean or Black British	3 (13.0)
Asian (Chinese, Indian, Bangladeshi, Pakistani, Other Asian)	3 (13.0)
Mixed or Multiple ethnic groups	1 (4.3)
Other	1 (4.3)
Role	
Lived experience of Long COVID	6 (26.1)
Clinician	10 (43.5)
Researcher	4 (17.4)
Regulator	2 (8.7)
Other	1 (4.3)

##### Small group discussions

All three groups included people from varied ethnic and professional backgrounds. Each of the three groups began by expressing a preference for pacing. As the conversation went on within each group, several potential interventions were discussed, including fatigue, breath work and heart rate monitoring.

Group 1 (n = 9): There was clear consensus on pacing being an important intervention as it was thought to address a number of different symptoms (particularly common symptoms such as breathlessness, brain fog and fatigue) and support return to work. However, there was recognition that pacing is challenging. The group also suggested that fatigue was a key symptom to address as it is the most common symptom of Long COVID, it is “hidden” and there is often associated stigma.

Group 2 (n = 5): There was discussion about Long COVID being multifaceted with fluctuating symptoms. Therefore, Group 2 considered that pacing and breathwork should be part of a wider intervention to address multiple symptoms, rather than a single-symptom intervention. Preferences were also voiced for the clinical decision support tool, which group members felt could provide tailored support to patients’ symptoms. Participants also supported evaluating the trial platform, which has the flexibility to evaluate different interventions and multiple approaches.

Group 3 (n = 9): Participants expressed a general preference for pacing, particularly alongside the use of heart rate monitors. Pacing was considered relatively safe, but participants noted a lack of high-quality support tools. Breathwork was also considered important; however, some clinicians felt that self-management strategies were only effective up to a certain point, beyond which healthcare provider support was needed. There was also recognition that Long COVID is a multi-system condition, that a trial platform would have the benefit of being able to test multiple interventions that could be targeted to patient needs.

Following the whole group discussion after the first vote, participants highlighted the importance of testing the electronic trial platform to allow future evaluation of multiple interventions to support those with Long COVID. Numerous existing pacing interventions were available and workshop participants argued that testing the trial platform could be of most benefit to facilitate long term research in this area and that pacing could be used as an exemplar.

##### Voting: round 1

**Fig. 2:**
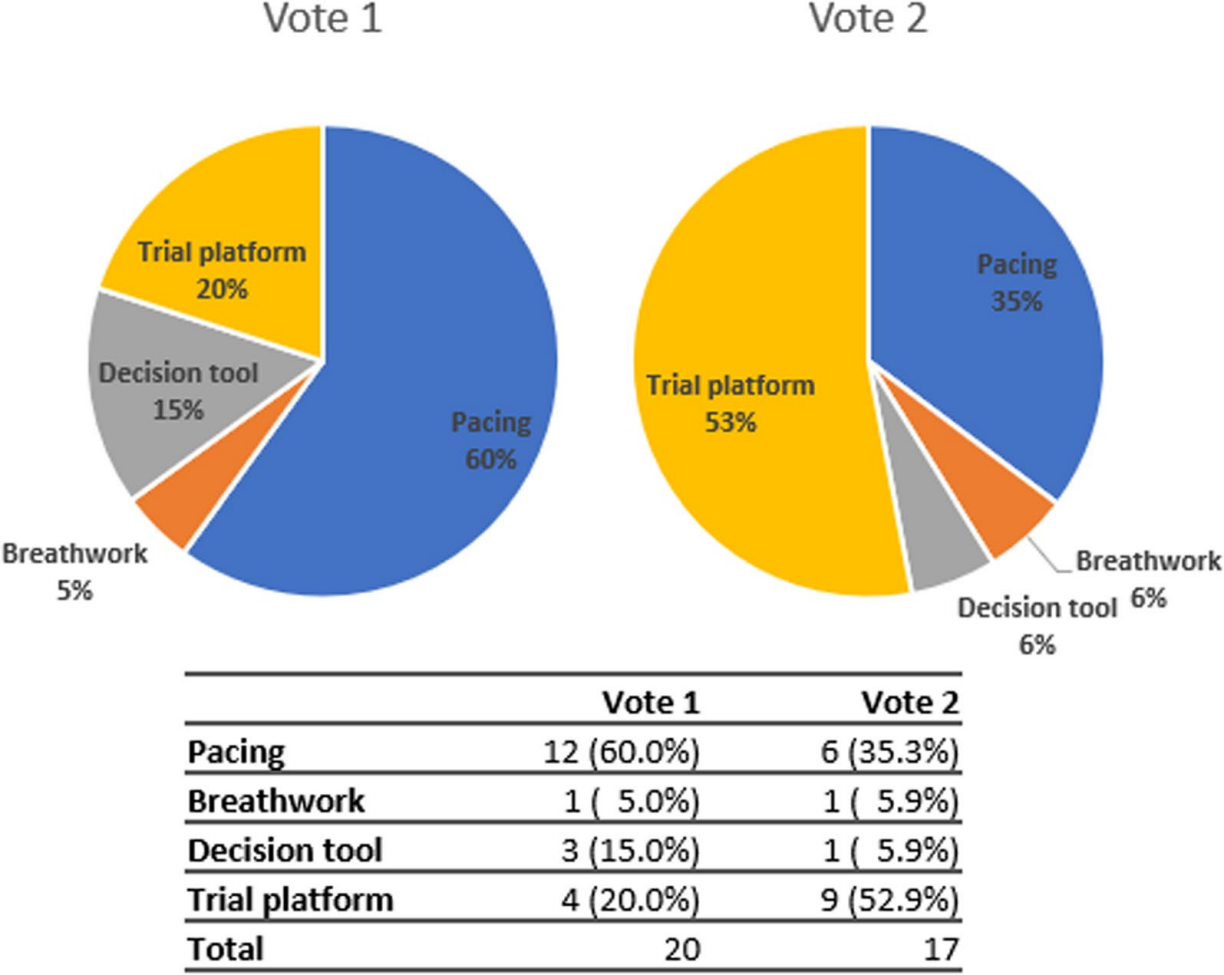
Consensus meeting voting results

Twenty participants voted in round one. The majority (60%; 12/20) voted for pacing (Fig. 2).

##### Whole group discussion

Pacing received the majority vote in round 1; however, the group discussed that Long COVID is a multi-system condition and that there is a requirement for rehabilitation to be multifaceted to address multiple symptoms and health needs. Some participants were interested in how wearables could be used to support pacing and track symptoms. There was discussion about self-management versus supported self-management (self-management delivered through a device), and participants with lived experience of Long COVID were in favour of receiving support from healthcare providers. Some individuals felt that a decision support tool would be useful for primary care clinicians. The group discussed and agreed that a trial platform is a unique opportunity to explore a range of interventions and could also be used as a template to test interventions in other conditions.

##### Voting: round 2

Seventeen participants voted in round two. The majority vote shifted to developing and evaluating the trial platform (52.9%; 9/17; Fig. 1). Based on the round 2 vote, developing and evaluating the trial platform was selected as the option to prioritise for the final phase of the TLC Study. As there was also a preference for evaluating interventions to support pacing, particularly from participants with Long COVID, it was agreed to focus the trial platform on testing different pacing interventions and resources.

#### Stage 2: co-selection of interventions

The survey and searches identified 35 resources for pacing (including resources for Long COVID and other long-term conditions), which were reduced to 25 following initial review from patient partners and the research team. Patient partners decided to include a mix of media formats (e.g., video, mobile application, information leaflet, etc.). Resources were selected based on content (considering factors such as simplicity, ease of understanding, and length/time taken to read or go through the resource), functionality (usability), aesthetics (visual appeal, layout, graphical quality), cost and availability. Three resources were selected: video, mobile application (app), and book (Table 2). The application and the video were free to use. The Pocket book of pacing was £5.50. All resources were in English.

**Table 2:** Summary of selected pacing resources

Resource	Population developed for	Developer	Description
Application: Spoonie Day (https://www.spoonieday.com/)	General population	Blackburn Labs	Activity/ symptom tracker Based on Spoon Theory, the app helps people track and monitor daily activities to see how they affect mood and energy levels
Video: The Why, When and How of Pacing: Long COVID's Most Important Lesson (https://www.youtube.com/watch?v=gUPvNwvkOlA)	Long COVID	Gez Medinger (lived experience of Long COVID)	50-min video about pacing, including information about what pacing is, the energy envelope, heart rate monitoring, difficulties in practice, rest, and practical tips
Information: The Pocket Book of Pacing (https://stickmancommunications.co.uk/product/the-pocket-book-of-pacing/)	Long-term conditions/disability	Stickman communications	Pocket-sized book containing information on pacing, including how it works, how it can be applied in real life and practical tips. Information is presented in short sentences with stickman images. Only available in hard copy

Originally, an information sheet on pacing with a heart rate monitor, designed by the Workwell Foundation [16], was also selected by patient partners. We sent the patient partners a Smart Watch to trial the pacing with heart rate monitor information sheet. They provided mixed feedback about the device, summarised in Table 3. Overall, there were concerns that features in the device which encouraged patients to exercise may not be appropriate in the context of Long COVID as patients highlighted the risks of overexertion and the negative impact of post-exertional malaise, which posed potential safety concerns. This resulted in a consensus-based decision to not include the Smart Watch with heart rate monitor as an intervention in the feasibility study (Table 3).

**Table 3:** Summary of feedback from patient partners on Smart Watch and heart rate monitor information sheet

Positives	Negatives
Heart rate/minute function is useful—the beeps warn of abnormal heart rate, leading the user to stop and rest	Device needs regular charging (every three days)
Stress level on body is easily monitored—if user becomes stressed, device tells them to breath in/out	Device is not designed for people with Long COVID—it often encourages the user to do exercise instead of slowing down
Device facilitates communication with GP—users can show some of the outcomes to their GP, which then can sometimes lead to referral to specific services	Device oxygen levels are lower than oxygen levels on a pulse oximeter. This can create anxiety amongst users who are unsure what to do about low readings and whether to seek medical help
Slim and light, user-friendly, easy to set up	Device may be too large for a user’s wrist and can sometime irritate the skin

### Work package 2: Co-design of the feasibility study

Co-design of the feasibility study occurred between March to July 2022.

#### “Experts by experience”: insights from lived experience

Through weekly meetings with the research team, our patient partners provided insights on their lived experience of long COVID and perspectives on research design, planning and decision making. Their real-life examples and stories were essential for shaping the design of research processes and educating the research team. For example, this included sharing the significant impact of fatigue on daily living, such as the need to rest for as long as a day after everyday activities such as bathing. This was particularly important as Long COVID is a new condition with limited scientific and clinical literature and expertise. Furthermore, our patient partners represented a range of age groups, ethnicities and personal circumstances which provided different perspectives and considerations.

#### Collectively design research processes

Patient partners were involved in decision making for all aspects of the design and delivery of the feasibility study. Examples of their input are summarised in Table 4.

**Table 4:** Summary of patient partners input into the design and delivery of the feasibility study

	Discussions/feedback	Impacts and outcomes of coproduction
Study objectives	Expressed preferences to evaluate pacing resources Utility of selected resources Practical approaches to pacing	Informed the following study objectives: explore pacing interventions, acceptability and adherence assess overall intervention fidelity
Eligibility criteria	Should eligibility criteria be restricted to fatigue given that pacing can positively impact other symptoms? Concluded that pacing may not be relevant to everyone with Long COVID since there are symptoms that pacing does not address (e.g., anosmia) Eligibility screening needs to be brief and capture fatigue that impacts daily activities	Included a single screening question for fatigue as part of the eligibility criteria
	Suggested translating recruitment and study materials to increase inclusivity of the study	After exploring options for translation, we were unable to meet this request as the intervention resources were all in English and some were not possible to translate such as the app Although translation was considered during intervention selection, most intervention options were only available in English The team agreed this was an important consideration for future work
Recruitment	Primary recruitment strategy was advertisements on social media Recognised that this strategy was not sufficiently inclusive and there is a need for additional approaches to recruitment in the community	A community recruitment strategy was developed based on suggestions from patient partners, including local radio, community groups (both Long COVID and general) and community organisations such as churches Patient partners reached out through their local networks and contacts (both Long COVID and general) In addition, the social media advertisement was edited to include “do you know anyone with Long COVID?” to increase reach through family/friends A question was included to identify where people were recruited from as part of the feasibility outcomes
Data collection	Conducted usability testing of the Aparito Atom5™ platform	Provided feedback on the functionality and design of the platform
Outcomes	Selecting a primary outcome for a definitive trial was discussed Expressed the importance of quality of life, which was considered more important than a fatigue score	Multiple patient-reported outcome measures were selected, including symptom burden [17], quality of life, fatigue and psychological impairment One of the objectives of the feasibility study will be to select a primary outcome for a definitive trial using both qualitative and quantitative data (e.g., data completeness)
Intervention selection	See work package 1: potential pacing resources were reviewed and tested	A decision was made to include a range of media formats (e.g., video, app, book) The three pacing resources that formed the intervention arms were all selected by patient partners
Control arm	There is no “usual care” for Long COVID. Therefore, patient partners were consulted on defining the comparator (control) arm	It was agreed to provide the control group with a link to the NHS website “Your COVID recovery” (https://www.yourcovidrecovery.nhs.uk) as this was considered the closest to “usual care”

#### Design and content of study materials

In some instances, participant facing documents such as participant information sheets were drafted by the research team and feedback was provided by patient partners. In some cases, patient partners had a more leading role in drafting content, such as instructions for the pacing intervention and design of recruitment materials. In particular, patient partners informed the content of the health economics questionnaire and intervention feasibility questionnaire. For example, patient partners suggested including an option for uploading pictures of medication boxes in the resources use questionnaire, as writing the names of medication is usually difficult and time-consuming. Frequently, discussions in meetings with the research team resulted in questions being added to the qualitative interview topic guide to be used at the end of the feasibility trial.

#### Testing the feasibility study platform (Atom5™)

The feasibility study used a digital platform, called Aparito Atom5™, for consent, randomisation, intervention delivery and data collection. Atom5™ can be accessed through an online web portal or an app (https://www.aparito.com/platform/). Patient partners conducted usability testing of the platform and provided feedback on functionality and design, which included issues such as:

- using more lay friendly terms, for example changing “Randomisation arm” to “Study pacing interventions”,
- advising the research team to programme notifications on the Atom5™ platform to prompt participants to access the intervention,
- identifying and fixing faulty weblinks.

### Work package 3: knowledge transfer

In addition to supporting planned knowledge transfer of academic outputs, ideas from patient partners resulted in several unplanned outputs, which were led by patient partners and supported by the research team.

Through involvement in the TLC study, patient partners benefitted from listening to each other’s experiences and coping strategies. They were keen to share their experiences of living with Long COVID and their involvement in research with the wider community. As a result, we created a series of videos where patient partners talked about living with Long COVID and shared tips on how they adapted to their new life. They were passionate about delivering a positive message, sharing tips and advice and ensuring outputs were accessible (such as including transcripts of videos). Videos were hosted on the TLC website and were shared on social media and Long COVID support groups. Patient partners also created infographics for key messages from the videos.

Patient partners also contributed to the TLC study webinar series whereby they led a webinar about "Lived experiences of Long COVID and collaboration on the TLC study". To reduce the burden, videos for the webinar were pre-recorded and patient partners answered questions live.

In terms of knowledge transfer related to the feasibility study, patient partners will co-design lay dissemination strategies and support the research team to interpret findings and identify key messages of importance to patients and the public. Their involvement in communicating the value of the study findings is particularly important as feasibility studies explore feasibility and acceptability, rather than intervention effectiveness.

### Work package 4: evaluation and reflections

#### Benefits to patient partners and the research team

##### Emotional benefit

Both the patient partners and research team members found the co-production process enjoyable, and they reported looking forward to coproduction meetings. Patient partners reported that they valued the opportunity to do something “useful” and they felt “heard and valued” by the research team. One patient partner commented that contributing to the coproduction process helped them deal with their experience of having Long COVID by helping them to feel useful on their journey to returning to work.

##### Knowledge

The research team benefitted greatly from insights shared by patient partners on their lived experience, which directly impacted on decision making and study design. This was particularly important as Long COVID is a novel condition and there is limited scientific evidence or literature. Even more importantly, they were able to provide critical insight into the day-to-day impact and emotional burden of living with Long COVID. Some research team members reported these personal stories fuelled their motivation and passion for the research.

Patient partners enjoyed learning about the research process and wider COVID-19 research from the research team. In particular, they gained knowledge about pacing through selecting and trialling the intervention pacing resources. Some thought that they understood pacing prior to their involvement, but learnt a lot more though the co-production experience, including one member who is a physiotherapist.

##### Peer support

Patient partners reported benefiting significantly from the opportunity to speak to other people with Long COVID and share experiences and tips on self-management. Some felt it increased their self-esteem and confidence. One patient partner commented “If I wasn’t in this group, I would be totally lost”. Others missed having the meetings when they were paused over the summer because of academic holidays and other commitments. The patient partners shared contact details with each other and communicated outside of the TLC study meetings. They have met once online outside the study and are planning to keep in touch with each other beyond the project end date.

#### Contextual factors

##### Group composition and dynamics

Both patient partners and researchers commented that the group “gelled well” early on and felt there was mutual respect and no conflict. Patient partners represented a mix of ages, ethnicities, and experiences, which enhanced knowledge and insight from different perspectives. The patient partners were all relatively “research naive”, which the research team considered beneficial as it created a natural, organic approach to involvement. Formal training was not provided, but instead relevant study processes were explained at relevant time points; for example, ethical review, and trial design considerations. Patient partners commented that they liked this approach and found it manageable.

For meetings with the research team, there was approximately equal representation between patient partners and members of the research team. This may have contributed to a healthy power dynamic and increased confidence for patient partners to contribute to discussions. Furthermore, the chair ensured patient partners were actively involved in discussions throughout the coproduction process. One member of the research team was a clinician and on rare occasion, patient partners had clinical questions that they were struggling to resolve with their own healthcare providers. It was therefore important to reiterate and delineate the role of the clinical academic as primarily being a researcher rather than as a clinician in the research context.

##### Impact of Long COVID symptoms

Long COVID significantly impacted on the daily lives of all patient partners and symptoms were unpredictable and fluctuated. All involvement in coproduction was conducted remotely and the research team worked with patient partners to be as inclusive and flexible as possible to maximise opportunities for involvement. For example, all meetings were optional, a break after 30 min was included in all meetings, the option to turn cameras off (which caused less fatigue) was always open, and patient partners were encouraged to do whatever was needed to reduce fatigue burden from the meetings, such as to lie in bed or just observe meetings and not actively contribute if they were feeling sufficiently well.

The research team did not require or expect that patient partners would attend all weekly meetings. The patient partners reported that they valued the meetings and benefitted from attending them, and therefore attended them regularly. However, some members of the research team were concerned that patient partners had attended at times when they were unwell or very fatigued (because they wanted to continue helping the research team). Furthermore, the research team had some concerns about the emotional burden of sharing personal and emotive experiences of living with Long COVID. In addition, the fluctuating symptoms of Long COVID resulted in some meetings being more productive than others.

##### Technology enabled involvement

All the coproduction meetings were conducted remotely, which was essential to enable the level of participation in coproduction from the patient partners. Virtual meetings reduced the burden on patient partners as they were able to join from the convenience of their home (which included lying in bed if necessary), involvement was restricted to one-hour meetings with a break (i.e. with no need for travel times) and the virtual conferencing software functions reduced fatigue (for example by enabling the camera to be turned off when a break was needed) and encouraged participation (e.g., chat box, virtual raising of hand). Remote meetings enabled a dialogue between people to be held without geographical limitations. Furthermore, from an administrative perspective, remote meetings were easier to organise than face-to-face meetings (e.g., not having to book a meeting room or arrange transport). Some patient partners initially needed support to access virtual meetings and understand the software functionality; however, they gained confidence over time through regular use of the technology.

#### Process factors

##### Administrative support

We were fortunate to have administrative support to arrange meetings, take meeting minutes and process payments for the patient partners. Without this support we would not have been able to meet as frequently as we did. Furthermore, having meeting minutes was considered beneficial by the research team to keep a record of decision making, for transparency and for accountability of the research team to deliver on what was discussed in each meeting.

##### Frequency of meetings

Weekly meetings were beneficial for relationship building and rapid decision making. Patient partners met twice a week with the first meeting being with several members of the research team and the second with a single researcher to facilitate discussions. Patient partners reported that they enjoyed having both meetings and benefitted differently from each. Although patient partners found the meetings tiring, they expressed a preference to meet bi-weekly. However, this incurred increased cost, time, and administrative burden but both members of the research team and patient partners felt this was outweighed by the benefits of these meetings.

## Discussion

This paper reports the process we used to co-produce a feasibility study to evaluate a platform for Long COVID trials, using pacing interventions as an exemplar. A stakeholder consensus workshop was first held for research prioritisation. Subsequently, patients were involved, as equal partners, in research prioritisation, intervention selection, co-design of feasibility study processes and study materials, and knowledge transfer. We used a flexible approach whereby co-production evolved organically and adapted to suit the needs and dynamics of the group. Through weekly contact with the research team, patient partners provided insights on their lived experience in conjunction with their perspectives on the feasibility study research design and project decision making. Co-production was effective and influenced important aspects of the study.

### Benefits and successes of co-production

The value of including patient partners and the public as equal partners in research design, rather than just being passively involved, is being increasingly recognised [18, 19]. Long COVID is a new condition that is poorly understood. Insights from patient partners with lived experience were therefore critical given the lack of scientific literature and expertise on effective therapies [7]. This was particularly important for understanding the impact of fatigue for people living with Long COVID. Although common in many long-term conditions, fatigue has different mechanisms and is experienced differently between conditions [20]. The insights of the patient partners on living with Long COVID fatigue were crucial in selecting interventions and, in the case of the heart rate monitor, identifying potential harms. As such, the research team respected and valued the contribution of patient partners as “experts by experience” [21] and considered their perspectives of equal importance to those of other research team members.

Co-production was mutually beneficial for both the research team and patient partners. Patient partners benefitted from feeling valued and useful, which was felt to be even more significant given that some were unable to work due to their Long COVID symptoms. Peer support was highly valued by patient partners and led them to communicate with each other outside of the TLC research meetings. The level of engagement from patient partners demonstrates how much they valued their involvement and their commitment to the project.

Regular (bi-weekly; i.e. twice a week) meetings were likely to be an important factor contributing to our successful co-production process. This enabled rapid building of relationships, confidence, and trust. It also facilitated shared decision making and collaborative working through ongoing dialogue between patient partners and the research team. Technology to enable remote video conferencing was essential for the frequency of meeting in terms of burden on patient partners, administrative time and travel expenses [22] Furthermore, the study was appropriately funded for this level of involvement and administrative support was essential.

Our guiding principles of co-production were informed by the NIHR INVOLVE guidance [9, 10] The research team recognised and respected the importance of lived experience perspectives and valued this insight for guiding decision making, such as intervention selection. An important principle was sharing of power, and respecting and valuing the knowledge of all members. We also aimed to be as inclusive and flexible as possible to accommodate and support the needs of the patient partners, through practical measures such as including a break in meetings to help prevent the onset of fatigue and supporting the use of virtual conference software. In addition to payment for their time and co-authorship of research publications, patient partners benefitted from their involvement through reciprocity in terms of feeling valued and building confidence and self-esteem. The team built and maintained relationships well early on in the coproduction process, which was reinforced by regular meetings and frequent dialogue.

### Challenges of co-production

Time commitment was one of the biggest challenges to our approach to successful co-production. The weekly frequency of meetings was driven by the tight timeframes for co-production, such as obtaining ethical approval and recruitment, and the subsequent need for rapid decision making. However, this frequency was partly maintained due to the expressed preferences of patient partners and unplanned outputs, such as the ‘living with Long COVID’ videos. There were some instances where patient partners had great ideas for outputs and public engagement which were unfortunately not feasible to deliver within the timescales and budget. This level of involvement also had cost implications as patient partners were paid an honorarium for their involvement. Our study had the appropriate budget to do this, but this may not be achievable in smaller projects.

The burden on patient partners is an important consideration, particularly in the context of Long COVID where symptoms can fluctuate and significantly impact on daily living, are unpredictable and fluctuate. Furthermore, the emotional burden of sharing personal experiences should not be underestimated. Although the research team made efforts to minimise this burden and placed no strict requirements or expectations for the level of involvement patient partners committed to, there were several occasions where their level of commitment to the study was such that they attended meetings at times when experiencing significant symptoms. It is also important to ensure patient partners are well enough to attend meetings and clarify the roles of the research team, particularly for academic clinicians, where there is a need to clearly delineate research from clinical roles. This was also highlighted by one of the study’s Principal Investigators who was also an academic clinician.

### Strengths and limitations of our co-production

A strength of our co-production work is that patient partners were involved early at the research prioritisation stage and will continue involvement through to the dissemination of feasibility study findings. The TLC research team agreed the guiding principles of co-production at the start of the process and were committed to upholding these principles. The team value co-production research and respected the knowledge and skills of patient partners. The TLC study was adequately costed to ensure sufficient funding for meaningful co-production, both in terms of payment for patient partners and capacity for the research team. Regular meetings with patient partners and the research team enabled us to build and maintain relationships, ongoing dialogue, rapid joint decision-making, and continuous reflection.

A limitation of our approach to co-production is that it was exclusively remote (i.e., not face-to-face) and predominantly digital through virtual conferencing software and by email correspondence. Although this approach has important advantages particularly for reducing the burden on patient partners, we risked excluding people without access to the internet or digital access. However, our patient partner group was diverse in terms of age, ethnicity, employment and Long COVID experience. Finally, although on occasion we did meet with patient partners outside usual business hours, most co-production involvement took place during working hours, which supported its sustainability.

Patient partners will be involved in the evaluation of the feasibility study in terms of interpretation of the data, co-authorship of the manuscript and dissemination.

## Conclusion

This paper reports the process we used to co-produce a feasibility study of pacing interventions for Long COVID. Co-production was effective and influenced important aspects of the study. In the context of Long COVID, insights from lived experiences were particularly crucial given the novelty of the condition, limited scientific evidence, including our systematic review [7] and expertise. Co-production was mutually beneficial for both the research team and patient partners.

## Data Availability

The datasets used and/or analysed during the current study are available from the corresponding author on reasonable request.

## Abbreviations

WHO: World Health Organisation
PASC: Post-acute sequelae SARS-CoV-2
NIHR: National Institute for Health and Care Research
TLC: Therapies for Long COVID
PPIE: Patient and public involvement and engagement
